# The Rényi Entropy and Entropic Cosmology

**DOI:** 10.3390/e28040467

**Published:** 2026-04-20

**Authors:** S. I. Kruglov

**Affiliations:** 1 Department of Physics, University of Toronto, 60 St. Georges St., Toronto, ON M5S 1A7, Canada; kruglov@rogers.com; 2 Canadian Quantum Research Center, 204-3002 32 Ave., Vernon, BC V1T 2L7, Canada

**Keywords:** entropic cosmology, Rényi entropy, dark energy, Friedmann equations, deceleration parameter, teleparallel gravity

## Abstract

Entropic cosmology with the Rényi entropy of the apparent horizon $S_{R}=\left(1/\alpha \right)\ln\left(1+\alpha S_{BH}\right)$, where $S_{BH}$ is the Bekenstein–Hawking entropy, is studied. By virtue of the thermodynamics–gravity correspondence, a model of dark energy is investigated. The generalized Friedmann equations for the Friedmann–Lemaître–Robertson–Walker spatially flat universe with barotropic matter fluid are obtained. We compute the dark energy density $\rho_{D}$, pressure $p_{D}$, and the deceleration parameter *q* of the universe. At some model parameters, the normalized density parameter of the matter $\Omega_{m0}\approx 0.315$ and the deceleration parameter $q_{0}\approx -0.535$ for the current epoch, which are in the agreement with the Planck data, are found. Making use of the thermodynamics–gravity correspondence, we describe the late-time acceleration of the universe. The entropic cosmology considered here is equivalent to cosmology based on the teleparallel gravity with the definite function F(T). The Hubble parameters are in approximate agreement (within 5 percents) with the observational Hubble data for redshifts 0.07≤z≤1.75 at the entropy parameter $\alpha \approx 0.305\;GH_{0}^{2}$.

## 1. Introduction

Data from observations of type Ia supernovae (SNe Ia) [1,2] and the cosmic microwave background (CMB) anisotropies [3], and baryon acoustic oscillations (BAO) [4] have shown that the universe currently accelerates. Thus, observations provide evidence for a dark-energy component. The current universe acceleration can be explained by the introduction of the cosmological constant Λ in the Einstein–Hilbert action. Then, there will be the contribution to the energy density in the Friedmann equation. Such energy density, named the dark energy, leads to the current universe acceleration and large-scale homogeneity and isotropy. But according to the observational data, there is a deviation from such a scenario with the constant cosmological constant in favor of the dynamical cosmological constant. Thus, there are discrepancies in the determination of the Hubble constant from different sources (Cepheids, supernovae Ia, and CMB) [5,6,7,8].

There is another way to describe universe acceleration by exploring entropic cosmology based on the thermodynamics of the apparent horizon in space-time with the Friedmann–Lemaître–Robertson–Walker (FLRW) metric [9,10,11,12,13,14,15,16,17,18,19,20] because of a correspondence between gravity and thermodynamics. The entropy of black holes as well as the entropy of the apparent horizon in cosmology is a function of the horizon area, and the temperature is linked with the surface gravity [21,22,23,24,25,26]. The first law of apparent horizon thermodynamics allows us to obtain Friedmann equations because the apparent horizon for the FLRW spatially flat space-time presents a thermodynamic system [9,11,27,28]. Due to the long-range nature of gravity, nonadditive entropies [29,30,31,32,33,34,35,36] were studied and holographic dark-energy models were considered [37,38,39,40,41,42,43].

Here, we explore the Rényi entropy to study the entropic cosmology based on the modified Friedmann equations. The equation of state (EoS) for barotropic perfect fluid with p=wρ, where *p* is the matter pressure and ρ is the matter energy density, was utilized. We compute the dark-energy density and pressure and show that late-time universe acceleration occurs. For some model parameters, the normalized density parameter of the matter $\Omega_{m}\approx 0.315$ and the deceleration parameter $q_{0}\approx -0.535$ for the current epoch are obtained, which are in agreement with the Planck data. It is shown that the entropic cosmology considered is equivalent to cosmology within the F(T) teleparallel gravity with torsion. The values of the predicted Hubble parameters are in approximate agreement with the observational Hubble data for redshifts 0.07≤z≤1.75 within 5 percent.

We utilize units with $\hbar =c=k_{B}=1$.

## 2. The Apparent Horizon Thermodynamics and Friedmann Equations

Let us study the thermodynamics of the apparent horizon in a space-time with the FLRW spatially flat metric, which is given by

(1) $$ds^{2}=-dt^{2}+a{\left(t\right)}^{2}\left(dx^{2}+dy^{2}+dz^{2}\right),$$

where a(t) is a scale factor. In a space-time with the FLRW spatially flat metric, the apparent horizon radius coincides with the Hubble radius, which is the distance from an observer where the expansion of the universe causes objects to recede at the speed of light. The apparent horizon radius is defined for c=1 as

(2) $$R_{h}=\frac{1}{H},$$

where the Hubble parameter of the universe is $H=\dot{a}\left(t\right)/a\left(t\right)$ and $\dot{a}\left(t\right)=\partial a/\partial t$. To obtain the Friedmann equation within entropic cosmology, we consider the first law of apparent horizon thermodynamics, which is given by [26,27,28]

(3) $$dE=-T_{h}dS_{h}+WdV_{h},$$

where $W=\frac{1}{2}\left(\rho -p\right)$ is the work density, $E=\rho V_{h}=\left(4\pi /3\right)\rho R_{h}^{3}$ and ρ, *p* are the energy density and pressure of a matter, respectively. The apparent horizon temperature reads [11]

(4) $$T_{h}=\frac{H}{2\pi }\left|1+\frac{\dot{H}}{2H^{2}}\right|.$$

It was mentioned in [11] that the term with $\dot{H}$ in Equation (4) is very small. With the help of the continuity equation, which represents the conservation law

(5) $$\dot{\rho }=-3H\left(\rho +p\right),$$

and Equations (3) and (4), we obtain [35]

(6) $$\frac{H^{3}}{2\pi }{\dot{S}}_{h}=4\pi \left(\rho +p\right).$$

According to Equations (5) and (6), to obtain the Friedmann equation, we need the entropy function. Here, we utilize the Rényi entropy [31]

(7) $$S_{R}=\frac{1}{\alpha }\ln\left(1+\alpha S_{BH}\right),$$

where $S_{BH}=\pi R_{h}^{2}/G=\pi /\left(GH^{2}\right)$ is the Bekenstein–Hawking (BH) entropy. The Rényi entropy (7) represents the deformation of the BH entropy. To obtain the corrections to BH entropy, we use the series expansion of the entropy (7) for small values of $\alpha S_{BH}$, which is given by

(8) $$S_{R}=S_{BH}-\frac{\alpha S_{BH}^{2}}{2}+\frac{\alpha^{2}S_{BH}^{3}}{3}+O\left(\alpha^{3}\right).$$

Equation (8) shows that corrections to the BH entropy $S_{BH}$ decrease the $S_{R}$ entropy. A similar effect occurs in quantum gravity [44]. One can assume that entropy (7) mimics the quantum gravity corrections to $S_{BH}$ entropy. By making use of Equations (6) and (7), one obtains the generalized Friedmann equation

(9) $$\frac{\dot{H}H^{2}}{H^{2}+b}=-4\pi G\left(\rho +p\right),$$

where b=απ/G. The dimension of parameter *b* is the same as the dimension of $H^{2}$. In our units ($\hbar =c=k_{B}=1$) the value of $GH^{2}$ is dimensionless. Taking into account Equation (5) and integrating Equation (9), we find the second generalized Friedmann equation

(10) $$H^{2}-b\ln\left(\frac{H^{2}+b}{b}\right)=\frac{8\pi G}{3}\rho .$$

If α=0 (b=0) in Equation (10), we arrive at the Friedmann equation of general relativity. The second term in the left side of Equation (10) can be treated as a contribution of dark energy to the matter density ρ.

## 3. The Dark Energy Density, Pressure, and Deceleration Parameter

We represent Equation (10) in the standard form of Friedmann’ equation,

(11) $$H^{2}=\frac{8\pi G}{3}\left(\rho +\rho_{D}\right),$$

where the dark energy density is given by

(12) $$\rho_{D}=\frac{3b}{8\pi G}\ln\left(\frac{H^{2}+b}{b}\right).$$

**Figure 1:**
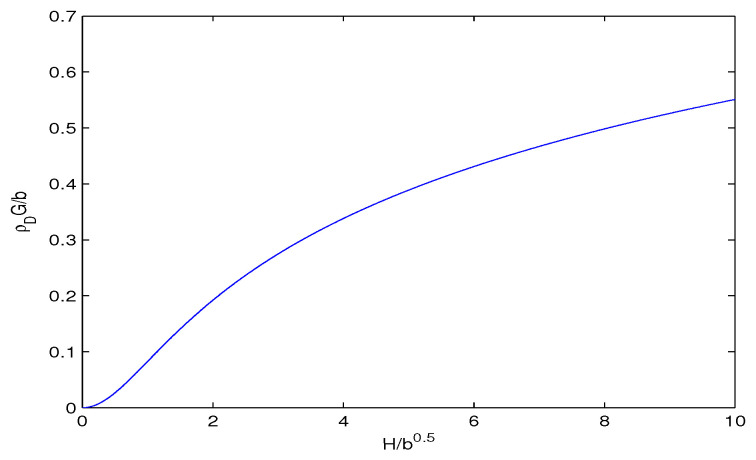
The reduced dark-energy density $\rho_{D}G/b$ vs. the parameter $H/\sqrt{b}$.

We plotted the dimensionless variable $\rho_{D}G/b$ versus $H/\sqrt{b}$ in Figure 1.

When $H/\sqrt{b}$ increases, the reduced dark-energy density $\rho_{D}G/b$ also increases and $\lim_{H\to 0}\rho_{D}=0$. As H→0 we have $R_{h}\to \infty$ which corresponds to the future era.

It is convenient to introduce the normalized density parameter of the matter $\Omega_{m}=\rho /\left(3M_{P}^{2}H^{2}\right)$ and the normalized density parameter of dark energy $\Omega_{D}=\rho_{D}/\left(3M_{P}^{2}H^{2}\right)$, where $M_{P}=1/\sqrt{8\pi G}$ is the reduced Planck mass. Then, from Equation (11), we obtain $\Omega_{m}+\Omega_{D}=1$. By the virtue of Equation (12), one obtains the normalized density for the matter and the normalized density parameter of dark energy, as follows:

(13) $$\Omega_{m}=1-\frac{b}{H^{2}}\ln\left(\frac{H^{2}+b}{b}\right],\;\;\;\Omega_{D}=\frac{b}{H^{2}}\ln\left(\frac{H^{2}+b}{b}\right].$$

Making use of the dimensionless variable $x=H^{2}/b$, Equation (13) becomes

(14) $$\Omega_{m}=1-\frac{1}{x}\ln\left(1+x\right),\;\;\;\Omega_{D}=\frac{1}{x}\ln\left(1+x\right).$$

**Figure 2:**
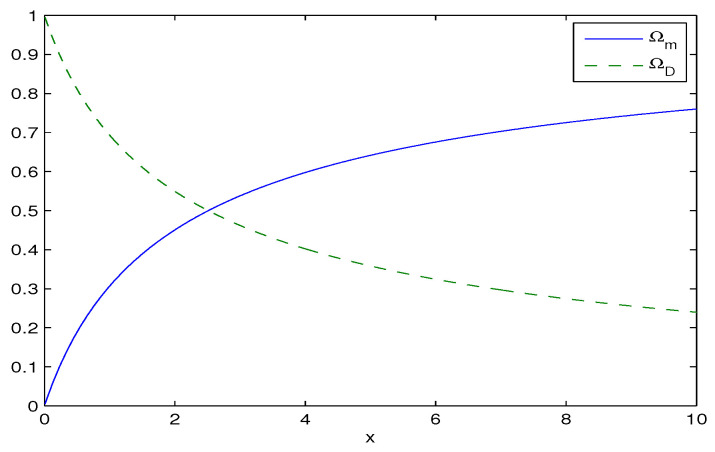
The normalized density parameters $\Omega_{m}$ and $\Omega_{D}$ vs. $x=H^{2}/b$.

The normalized density parameter of the matter $\Omega_{m}$ and the normalized density parameter of dark energy $\Omega_{D}$ are depicted in Figure 2.

According to Figure 2 as x→∞ (H→∞, $R_{h}\to 0$), we have $\Omega_{m}\to 1$ and $\Omega_{D}\to 0$ corresponding to the matter-dominated era. As x→0 (H→0, $R_{h}\to \infty$), $\Omega_{D}\to 1$ and $\Omega_{m}\to 0$ correspond to the dark-energy dominated epoch (the future era). The Planck data show that $\Omega_{m0}\approx 0.315$ [3] for the current era. The solution to Equation (14) for $\Omega_{m0}=0.315$ is given by

(15) $$x=\frac{1}{137}\left[-200W_{-1}\left(-\frac{137}{200e^{137/200}}\right)-137\right]\approx 1.04282,$$

where W(z) is the Lambert function, which obeys the equation Wexp(W)=z. The $W_{-1}\left(z\right)$ is the lower branch of W(z) for W(z)≤−1. Then, we obtain the entropy parameter

(16) $$\alpha =\frac{bG}{\pi }=\frac{GH_{0}^{2}}{1.04282\pi }\approx 0.305\;GH_{0}^{2},$$

where $H_{0}$ is the Hubble rate at the current time. To compute the EoS for dark energy $w_{D}$, we need the pressure $p_{D}$. We assume that the dark energy and pressure obey the continuity equation (Equation (5)), which gives

(17) $$p_{D}=-\frac{{\dot{\rho }}_{D}}{3H}-\rho_{D}.$$

By virtue of Equations (12) and (17), one finds the equation for the pressure corresponding to dark energy

(18) $$p_{D}=-\frac{b\dot{H}}{4\pi G(b+H^{2})}-\frac{3b}{8\pi G}\ln\left(\frac{H^{2}+b}{b}\right).$$

Making use of Equations (9), (10), and (18) and the equation of state EoS for barotropic matter fluid w=p/ρ, we obtain the pressure

(19) $$p_{D}=\frac{3b(1+w)}{8\pi GH^{2}}\left[H^{2}-b\ln\left(\frac{H^{2}+b}{b}\right)\right]-\frac{3b}{8\pi G}\ln\left(\frac{H^{2}+b}{b}\right).$$

From Equations (12) and (19), we find the EoS for dark energy $w_{D}=p_{D}/\rho_{D}$,

(20) $$w_{D}=\frac{1+w}{H^{2}}\left(\frac{H^{2}}{\ln\left(H^{2}/b+1\right)}-b\right)-1.$$

By using the dimensionless variable $x=H^{2}/b$, Equation (20) becomes

(21) $$w_{D}=\frac{1+w}{x}\left(\frac{x}{\ln(x+1)}-1\right)-1.$$

**Figure 3:**
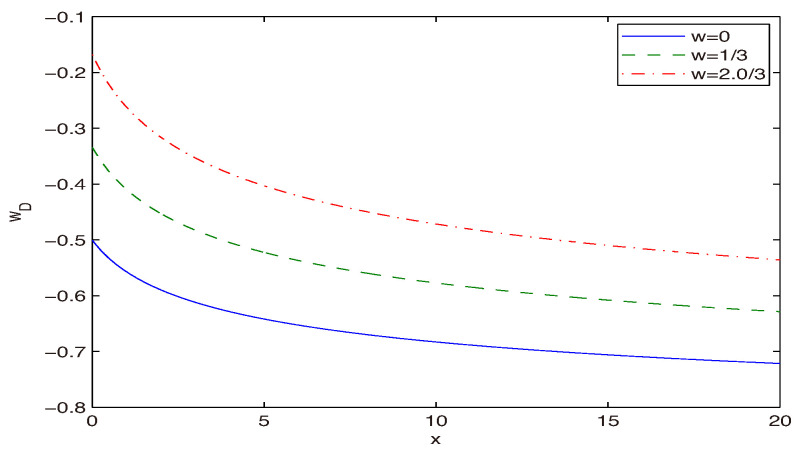
The EoS for dark energy $w_{D}$ vs. the dimensionless variable $x=H^{2}/b$.

The EoS for dark energy $w_{D}$ versus *x* is depicted in Figure 3.

By the virtue of Equation (21), one has $\lim_{x\to \infty }w_{D}=-1$ and $\lim_{x\to 0}w_{D}=\left(w-1\right)/2$. Thus, for the large Hubble parameter *H* (the small $R_{h}$), the dark-energy EoS is $w_{D}=-1$, which corresponds to the inflation era.

To analyze the observational data, it is convenient to introduce the redshift z=1/a(t)−1. Then, using the continuity Equation (5) and EoS p=wρ, we obtain the density energy of the matter in the form

(22) $$\rho =\rho_{0}{\left(1+z\right)}^{3(1+w)},$$

where $\rho_{0}$ is the energy density of the matter at the present time. From Equations (10) and (22), we obtain the generalized Friedmann equation as follows:

(23) $$H^{2}-b\ln\left(\frac{H^{2}+b}{b}\right)=\frac{8\pi G\rho_{0}}{3}{\left(1+z\right)}^{3(1+w)}.$$

To plot the Hubble parameter versus redshift, we find from Equation (23) the redshift

(24) $$z={\left[\frac{3}{8\pi \rho_{0}G}\left(H^{2}-b\ln\left(\frac{H^{2}+b}{b}\right)\right)\right]}^{1/(3(1+w))}-1,$$

and introducing dimensionless parameters $\bar{H}=H/\sqrt{G\rho_{0}}$, $\bar{b}=b/\left(G\rho_{0}\right)$, we represent Equation (24) as

(25) $$z={\left[\frac{3}{8\pi }\left({\bar{H}}^{2}-\bar{b}\ln\left(\frac{{\bar{H}}^{2}+\bar{b}}{\bar{b}}\right)\right)\right]}^{1/(3(1+w))}-1.$$

**Figure 4:**
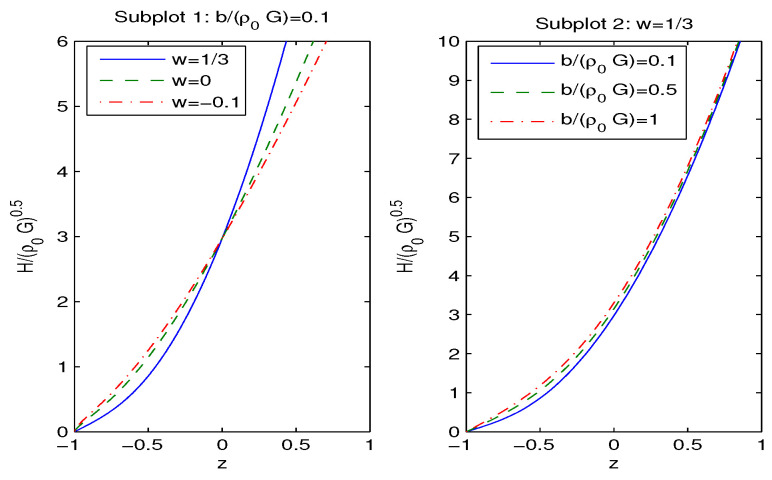
The reduced Hubble rate $\bar{H}$ vs. redshift *z*.

By the virtue of Equation (25), we depicted the reduced Hubble parameter $\bar{H}$ versus redshift *z* in Figure 4.

In accordance with Figure 4, when the redshift *z* increases, the reduced Hubble parameter $\bar{H}$ also increases. According to the left panel of Figure 4, when parameter *w* increases at fixed $\bar{H}$, the redshift *z* decreases at z>0. The right panel of Figure 4 shows that when parameter $\bar{b}$ increases at fixed z, the reduced Hubble parameter $\bar{H}$ also increases. At z=−1, we have H=0.

Now, we are going to fix the model parameter *w* to agree with the Plank data. For this goal, we consider the deceleration parameter, which is given by

(26) $$q=-\frac{\ddot{a}a}{{\dot{a}}^{2}}=-1-\frac{\dot{H}}{H^{2}}.$$

The acceleration phase of the universe occurs when q<0 and when q>0, the deceleration phase takes place. Making use of Equations (9), (22), and (26), we obtain the deceleration parameter as

(27) $$q=\frac{4\pi G\rho_{0}\left(1+w\right)\left(H^{2}+b\right)}{H^{4}}{\left(1+z\right)}^{3(1+w)}-1.$$

By virtue of Equations (23) and (27), we find

(28) $$q=\frac{3\left(1+w\right)\left(H^{2}+b\right)}{2H^{4}}\left(H^{2}-b\ln\left(\frac{H^{2}+b}{b}\right)\right)-1.$$

Using dimensionless variable $x=H^{2}/b$, Equation (28) becomes

(29) $$q=\frac{3(1+w)(1+x)}{2x^{2}}\left(x-\ln(1+x)\right)-1.$$

**Figure 5:**
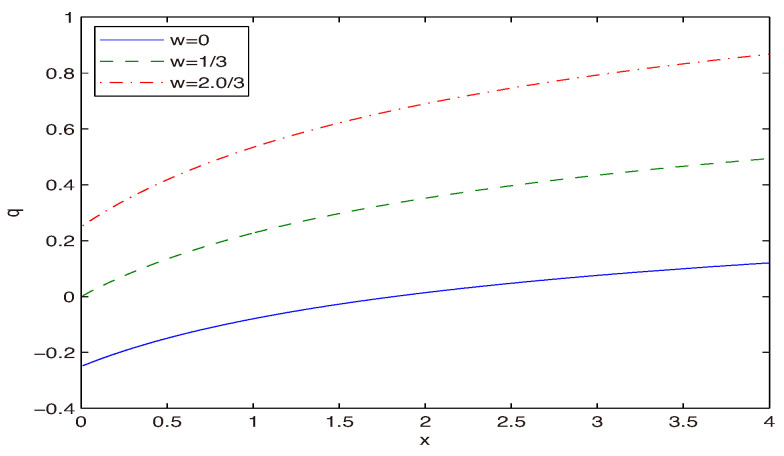
The deceleration parameter *q* vs. $x=H^{2}/b$ at w=0,1/3,2/3.

Making use of the value $x=H_{0}^{2}/b\approx 1.04282$ ($\alpha \approx H_{0}^{2}G/\left(1.04282\pi \right)$), this gives the normalized density of the matter field at the current time $\Omega_{m0}\approx 0.315$, and the deceleration parameter $q_{0}\approx -0.535$ [3], we obtain the solution to Equation (29) for the EoS parameter of the matter w≈−0.4976. We plotted the deceleration parameter *q* versus *x* in Figure 5.

In accordance with Figure 5, we have two phases, the universe acceleration and deceleration. Taking into account Equation (28), we obtain the asymptotic

(30) $$\lim_{H\to \infty }q=\frac{3w+1}{2}.$$

It follows from Equation (30) that when w>−1/3 (q>0) at large *H*, the universe decelerates and the universe accelerates at w<−1/3. The calculated value w≈−0.4976 is in agreement with this requirement. Using Equation (29) at q=0, we obtain the equation for the transition phase

(31) $$w=\frac{2x^{2}}{3\left(1+x\right)\left(x-\ln(1+x)\right)}-1.$$

We obtain limits of the EoS parameter *w* at x→0 and x→∞ as follows:

(32) $$\lim_{x\to 0}w=\frac{1}{3},\;\;\;\;\lim_{x\to \infty }w=-\frac{1}{3}.$$

**Figure 6:**
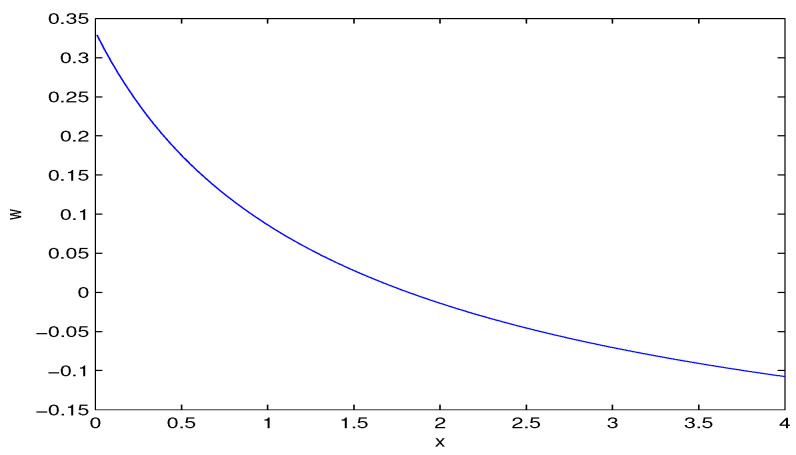
The EoS parameter for the matter *w* vs. $x=H^{2}/b$ at q=0.

We plotted the EoS parameter for the matter *w* versus *x* in Figure 6.

According to Figure 6, when $x=H^{2}/b$ increases, the EoS parameter *w* decreases. At a large Hubble parameter *H*, for q=0, w→−1/3 and at small *H*, we have w→1/3.

The entropy parameter is given by $\alpha =bG/\pi =GH^{2}/\left(x\pi \right)$. For the current era x≈1 and $\alpha \approx GH_{0}^{2}/\pi$. Because $GH_{0}^{2}\ll 1$, we have for the current era α≪1 and, therefore, quantum corrections to the Bekenstein–Hawking entropy $S_{BH}$, according to Equation (8), are small. When parameter *x* is small, the entropy parameter α will be large and the quantum effects are quite dominant. According to Figure 2, this happens when the normalized density parameter of dark energy approaches $\Omega_{D}\to 1$.

## 4. F(T)-Gravity from the Rényi Entropy

In the theory of Teleparallel Equivalent to General Relativity (TEGR), the curvature is replaced by torsion. In this self-consistent theory of gravity, the dynamics are the same as in general relativity. In TEGR, tetrad fields (vierbeins) define a basis that describes the geometry of space-time. Vierbeins define a torsion tensor which is the source of gravity presenting the antisymmetric contribution of the Christoffel connection. The torsion scalar *T* is constructed by the torsion tensor and defines the gravitational action. In F(T) gravity, the Lagrangian density of TEGR is modified by using an arbitrary function of the torsion scalar. In the teleparallel theory of gravity, the Weitzenböck connection is used, and the field equations are of the second-order. The torsion field *T* is given by [45,46]

(33) $$T={S_{\rho }}^{\mu \nu }{T^{\rho }}_{\mu \nu }.$$

The superpotential ${S_{\rho }}^{\mu \nu }$ and the contortion tensor ${K^{\mu \nu }}_{\rho }$ are

$${S_{\rho }}^{\mu \nu }=\frac{1}{2}\left({K^{\mu \nu }}_{\rho }+\delta_{\rho }^{\mu }{T^{\alpha \nu }}_{\alpha }-\delta_{\rho }^{\nu }{T^{\alpha \mu }}_{\alpha }\right),$$

(34) $${K^{\mu \nu }}_{\rho }=-\frac{1}{2}\left({T^{\mu \nu }}_{\rho }-{T^{\nu \mu }}_{\rho }-{T_{\rho }}^{\mu \nu }\right),$$

and the torsion tensor is defined as

(35) $${T^{\rho }}_{\mu \nu }=e_{i}^{\rho }\left(\partial_{\mu }e_{\nu }^{i}-\partial_{\nu }e_{\mu }^{i}\right),$$

where $e_{\nu }^{i}$ (i=0,1,2,3) is a vierbein field. In the flat metric of the tangent spacetime $\eta_{ij}$, the metric tensor is $g_{\mu \nu }=\eta_{ij}e_{\mu }^{i}e_{\nu }^{j}$. In FLRW metric (1), the vierbein field is $e_{\mu }^{i}=\mathrm{diag}\left(1,a,a,a\right)$ and the torsion scalar becomes $T=-6H^{2}$. The variation of the action with respect to $e_{\mu }^{i}$ with the Lagrangian F(T) gives the equation [47]

(36) $$\frac{1}{6}\left[F\left(T\right)-2TF^{\prime }\left(T\right)\right]=\left(\frac{8\pi G}{3}\right)\rho .$$

Making use of Equations (10) and (36) and $T=-6H^{2}$, we obtain

(37) $$F^{\prime }\left(T\right)-\frac{F(T)}{2T}=\frac{1}{2}+\frac{3b}{T}\ln\left(1-\frac{T}{6b}\right).$$

By integrating Equation (37) we find the equation as follows:

(38) $$F\left(T\right)=T-6b\ln\left(1-\frac{T}{6b}\right)+2\sqrt{-6bT}\arctan\left(\sqrt{-\frac{T}{6b}}\right),$$

where we use the integration constant to be C=0. We used the relation $i\;\tanh^{-1}\left(ix\right)=-\arctan\left(x\right)$ because $T=-6H^{2}<0$. Some teleparallel gravity models were studied in [48,49]. Thus, we showed that the entropic cosmology with the Rényi entropy (7) is equivalent to a cosmology based on the teleparallel gravity with the function (38).

## 5. Conclusions

We studied the entropic cosmology with the Rényi entropy $S_{R}=\left(1/\alpha \right)\ln\left(1+\alpha S_{BH}\right)$, which describes the dark energy and leads to the current acceleration of the universe. The spatial flat FLRW universe and the matter barotropic perfect fluid are implied. By the virtue of the first law of apparent horizon thermodynamics, we obtained the modified Friedmann equations, which include the density of dark energy. We assumed that the dark energy density $\rho_{D}$ and pressure $p_{D}$ obey the continuity equation (the conservation law). The EoS $w_{D}=p_{D}/\rho_{D}$ has been computed with $\lim_{H\to \infty }w_{D}=-1$, which shows that at $R_{h}\to 0$, the de Sitter space-time occurs and the inflation of the universe takes place. In the model under consideration, the universe may have two phases, acceleration and deceleration, due to the dark energy. We showed that at the entropy parameter $\alpha \approx 0.305GH_{0}^{2}$ and w=−0.4976 the deceleration parameter has the value $q_{0}\approx -0.535$ and the normalized density parameter of the matter is $\Omega_{m0}\approx 0.315$, which are in agrement with the Planck data at the current epoch [3]. It was shown that entropic cosmology studied can be considered as the cosmology based on the teleparallel gravity with the function F(T) obtained. The Hubble parameters are in approximate agreement with the observational Hubble data for 0.07≤z≤1.75 at the entropy parameter $\alpha =bG/\pi \approx 0.305\;GH_{0}^{2}$ (see Appendix A).

## Figures and Tables

**Figure A1:**
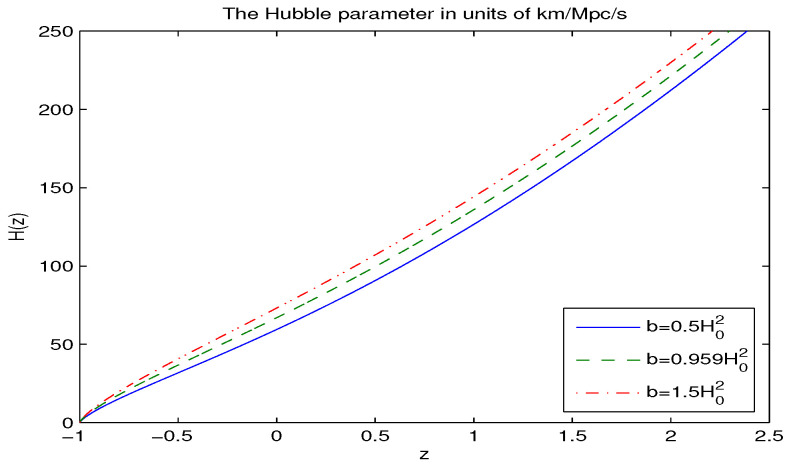
The Hubble parameter H(z) in units of km/Mpc/s vs. *z*.

## Data Availability

Data are contained within the article.

## Appendix A. The Hubble Parameter and Observational Data

Making use of Equation (10) and the energy density

(A1) $$\rho =\rho_{m}+\rho_{r}=\rho_{m0}{\left(1+z\right)}^{3}+\rho_{r0}{\left(1+z\right)}^{4},$$

where $\rho_{m}$ is the energy density of the non-relativistic matter in the form of dust (w=0) and $\rho_{r}$ is the radiation energy density (w=1/3), we obtain

(A2) $$H^{2}\left(z\right)-b\ln\left(\frac{H^{2}\left(z\right)}{b}+1\right)=H_{0}^{2}\Omega_{m0}{\left(1+z\right)}^{3}+H_{0}^{2}\Omega_{r0}{\left(1+z\right)}^{4}.$$

One can neglect the contribution of the radiation energy density for small redshifts because $\Omega_{r0}\approx 10^{-4}$. In accordance with Equation (A2) at z=−1 we have the value H(−1)=0. By the virtue of Equation (A2) with the current Hubble parameter $H_{0}=67\;\mathrm{km}/\mathrm{Mpc}/s$ and $\Omega_{m0}=0.315$, we depicted the Hubble parameter H(z) in units of km/Mpc/s in Figure A1.

According to Figure A1, the values of Hubble parameters are in approximate agreement with the observational Hubble data for 0.07≤z≤1.75 [50,51,52,53,54,55,56,57,58,59,60] at $b=\alpha \pi /G\approx 0.959\;H_{0}^{2}$ ($\alpha \approx 0.305\;GH_{0}^{2}$) which gives the correct value for the normalized density parameter of the matter at the current era. Some observational Hubble data for 0.07≤z≤1.75 are represented in Table A1.

**Table A1:** Observational Hubble data for 0.07≤z≤1.75. The Hubble parameter $H_{OHD}$ is in units of km/Mpc/s.

*z*	0.07	0.18	0.24	0.429	0.45	0.48	0.593	0.875	1.3	1.75
$H_{OHD}$	69	75	79.7	91.8	92.8	97	104	125	168	202
Ref.	[50]	[54]	[52]	[51]	[51]	[53]	[54]	[54]	[52]	[52]

Introducing the dimensionless parameter $E\left(z\right)=H\left(z\right)/H_{0}$ and making use of parameter $b\approx 0.959H_{0}^{2}$ which gives the normalized density parameter $\Omega_{m0}=0.315$, we represent Equation (A2) as follows:

(A3) $$E{\left(z\right)}^{2}\left(z\right)-0.959\ln\left(\frac{E^{2}\left(z\right)}{0.959}+1\right)=0.315{\left(1+z\right)}^{3}+10^{-4}{\left(1+z\right)}^{4}.$$

Solving Equation (A3) and by virtue of relation $H\left(z\right)=E\left(z\right)H_{0}$ with $H_{0}=67$ km/Mpc/s, we obtain the Hubble parameters for the interval 0.07≤z≤1.7 represented in Table A2. We include also in Table A2 the relative percentage deviation $R=100\left(H-H_{OHD}\right)/H_{OHD}$, which shows the deviation of observational Hubble parameters from the predicted Hubble parameters.

**Table A2:** The Hubble parameters (in km/Mpc/s) for 0.07≤z≤1.75, calculated from Equation (A3) with the relation $H\left(z\right)=E\left(z\right)H_{0}$, and the relative percentage deviation *R*.

*z*	0.07	0.18	0.24	0.429	0.45	0.48	0.593	0.875	1.3	1.75
*H*	71.4	78.4	82.2	94.7	95	98.2	106.0	125	159.8	198.5
*R*	3.43	4.47	3.17	3.20	2.37	1.25	1.96	0	−4.88	−1.76

Table A2 shows that the deviation of predicted values of Hubble parameters from the observational Hubble data are small and the relative percentage deviations $R=100\left(H-H_{OHD}\right)/H_{OHD}$ are within 5 percent.
